# Molecular characterization of *Toxoplasma gondii* Type II in sheep abortion in Sardinia, Italy

**DOI:** 10.1051/parasite/2014007

**Published:** 2014-02-19

**Authors:** Giovanna Chessa, Valentina Chisu, Rosaura Porcu, Giovanna Masala

**Affiliations:** 1 Istituto Zooprofilattico Sperimentale della Sardegna Via Duca degli Abruzzi, 8 07100 Sassari Italy

**Keywords:** *Toxoplasma gondii*, Genetic characterization, Sheep, Abortion

## Abstract

During 2009–2010, 161 tissue samples (142 placentas, 16 brains, and 3 livers) from aborted ovine fetuses on Sardinia Island, Italy, were tested for toxoplasmosis. Organs that showed a positive result by nested polymerase chain reaction (PCR) targeting the *ITS1* region for *Toxoplasma gondii* were also amplified with 11 genetic markers (*SAG1*, *5′-SAG2*, *3′-SAG2*, *SAG3*, *BTUB*, *GRA6*, *c22-8*, *c29-2*, *L358*, *PK1*, and *Apico*) and then subjected to PCR/RFLP for genetic typing. *T. gondii* DNA was found in 5 placentas, 14 brains, and 2 livers by PCR analysis and all isolates displayed Type II alleles at all 11 loci with all 11 markers. The results indicate that the Type II *T. gondii* is associated with ovine abortion.

## Abstract (fr)

Pendant la période 2009–2010, 161 échantillons de tissus (142 placentas, 16 cervelles et 3 foies) provenant de fœtus ovins avortés de Sardaigne, Italie, ont été testés pour la toxoplasmose. Les organes trouvés positifs par PCR visant la région ITS1 de *Toxoplasma gondii* ont été amplifiés avec 11 marqueurs génétiques (*SAG1*, *5′-SAG2*, *3′-SAG2*, *SAG3*, *BTUB*, *GRA6*, *c22-8*, *c29-2*, *L358*, *PK1* et *Apico*) et ensuite soumis à PCR/RFLP pour génotypage. De l’ADN de *T. gondii* a été trouvé dans 5 placentas, 14 cervelles et 2 foies par les analyses PCR et tous les isolats avaient des allèles de Type II pour les 11 loci avec tous les 11 marqueurs. Les résultats indiquent que *T. gondii* de Type II est associé avec les avortements ovins.

## Introduction

Since the 1950s, *Toxoplasma gondii* has been recognized as a common cause of abortions in sheep. Why some sheep abort whereas most do not is not fully understood. *T. gondii* is a single species in the genus but recent studies indicate that it has several genotypes, and some genotypes are more pathogenic for mice versus others [4]. However, nothing is known of this association in sheep [10].

The aim of this study is to characterize *T. gondii* from aborted ovine samples by PCR/RFLP analysis in order to understand the strains circulating in Sardinia, Italy.

## Materials and methods

### Samples of abortion products

Aborted ovine samples were submitted to the Istituto Zooprofilattico Sperimentale by practitioner veterinarians from farms located in different municipalities of Sardinia, Italy, during 2009–2010. In Sardinia, the primary sector is still of outstanding importance, especially sheep rearing; on the island there are 43,877 farms, of which 11,356 are sheep farms with a total of 3,279,420 sheep, corresponding to half of the total Italian stock (Reg. CE 1760/2000 – BDN data). The samples were from pastured sheep, therefore data on individual ewes were limited except that submitted fetuses were not twins. A total of 161 samples (142 placentas, 16 brains, 3 livers) from aborted fetuses were digested by using trypsin concentration as described by Masala et al. [8].

### DNA extraction and detection of *T. gondii* by PCR

DNA was extracted from digested tissues using the DNeasy Tissue Kit (Qiagen, Valencia, CA, USA) in accordance with the manufacturer’s instructions.

*T. gondii* infections were initially confirmed by nested PCR assays targeting the multicopy 18S-5.8S rRNA internal transcribed spacer (*ITS1*) region, as previously described by Hurtado et al. [6].

All PCR reactions were performed in a GeneAmp PCR System 9700 (Applied Biosystems, Foster City, CA, USA). A positive control (*T. gondii* TS-4, ATCC 40050) and negative control were included in all experiments. The expected size of the amplified DNA fragment was 227 bp. PCR products were resolved on a 1–1.5% agarose gel in 1× TAE buffer (0.04 m Tris-acetate, 0.001 m EDTA). After electrophoresis at 100 volts for 60 min, gels were stained with ethidium bromide and examined under UV light in an ImageMaster VDS-CL System (Amersham Biosciences Europe GmbH, Milano, Italy).

### Genotype analysis

The lineage type was performed by nested PCR amplification of eleven genetic markers: *SAG1*, *5′-SAG2*, *3′-SAG2*, *SAG3*, *BTUB*, *GRA6*, *c22-8*, *c29-2*, *L358*, *PK1*, and *Apico* [11], and thereafter was analyzed by restriction fragment-length polymorphism (RFLP).

For each genetic marker, the target DNA sequences were amplified by PCR using primers for individual markers. In brief, each nested PCR reaction was carried out in 25 μL of volume containing 1× PCR buffer, 25 mM MgCl_2_, 100 pmol/μL each of the dNTPs, 25 pmol/μL each of the forward and reverse primers, 0.5 units of AmpliTaq Gold Polymerase (Roche), and 1.5 μL of DNA extract. The reaction mixture was treated at 95 °C for 5 min, followed by 30 cycles of 95 °C for 1 min, 56 °C for 1 min and 72 °C for 2 min, and 72 °C for 10 min. PCR products were treated with restriction enzymes and resolved in 2.5% agarose gel by electrophoresis to reveal the RFLP patterns of the isolates.

Reference strains of *T. gondii* were also used in genotyping, including Type I TS-4 (ATCC 40050), Type II (Me 49), and Type III (VEG).

## Results

*T. gondii* DNA was found in 5/142 (3.5%) placenta samples, 14/16 (87%) brain samples, and 2/3 (66.6%) samples of ovine liver. Among the 14 positive brains, five belong to primiparous and eight to pluriparous sheep. Both positive livers analyzed belong to pluriparous sheep, while no data about the positive placentas were available.

The presence of Type II was detected in 5 placenta, 14 brain, and 2 liver samples at all loci.

## Discussion

This is the first attempt at genotyping *T. gondii* from sheep hosts in Italy. The data are based on DNA extracted directly from naturally infected tissues. For definitive studies DNA characterization from viable parasites is needed. Our results indicate the presence of clonal Type II *T. gondii* using recently developed 11 RFLP markers. Type II was also identified in aborted sheep from the UK [9] and Denmark [7], but their results were based only on the SAG2 locus. Type II *T. gondii* is the most prevalent in all hosts in Europe including adult sheep [3, 5, 11]. However, different atypical genetic types were prevalent in asymptomatic and diseased sheep in the Americas [1, 2].
